# Supplementary figures and images for: Variable effects of temperature on insect herbivory
Source: PeerJ. 2014 May 6;2:e376. doi: 10.7717/peerj.376 (PMC4017821; doi:10.7717/peerj.376)

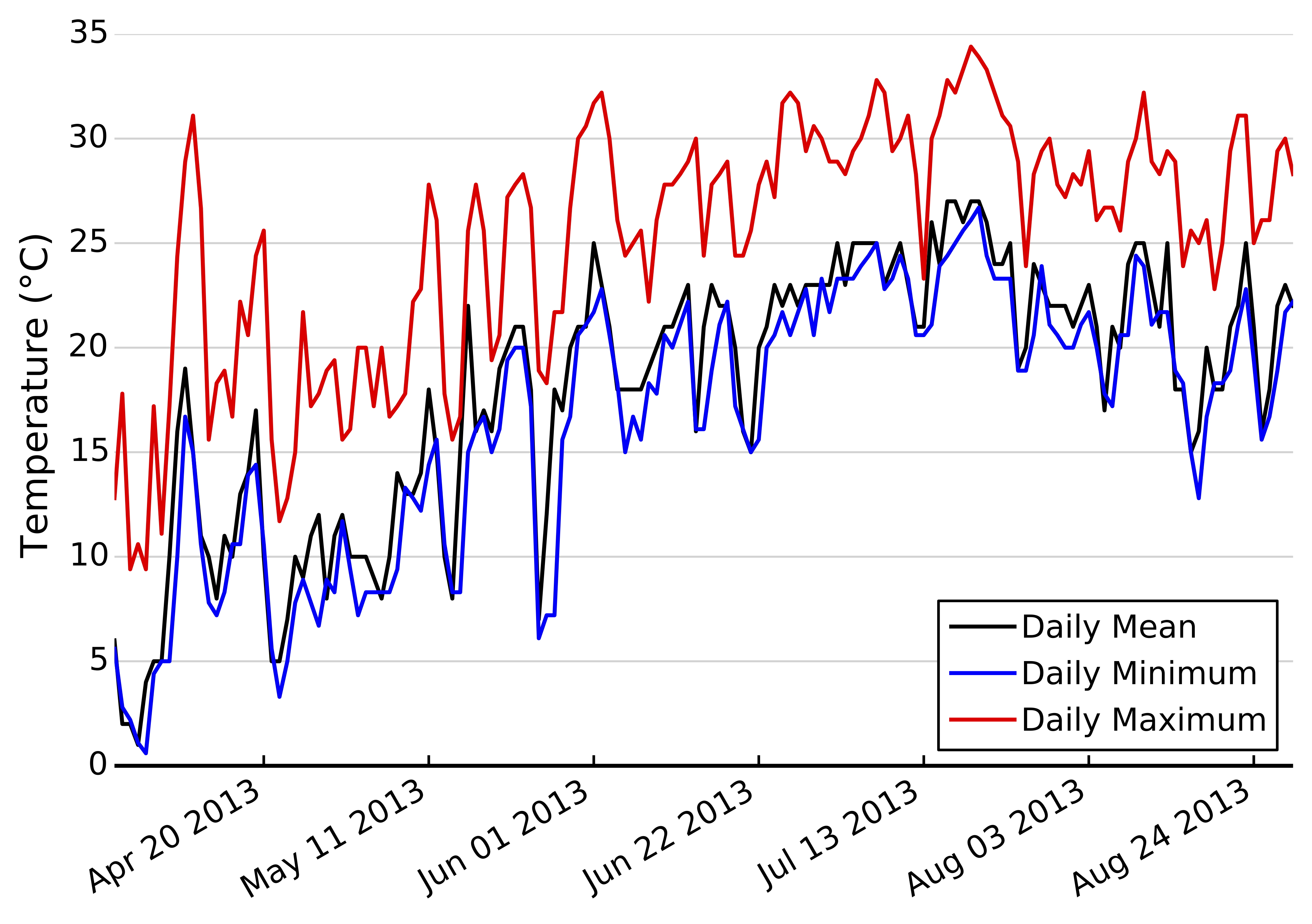

Supplement: Figure S1 [file peerj-02-376-s001.png]

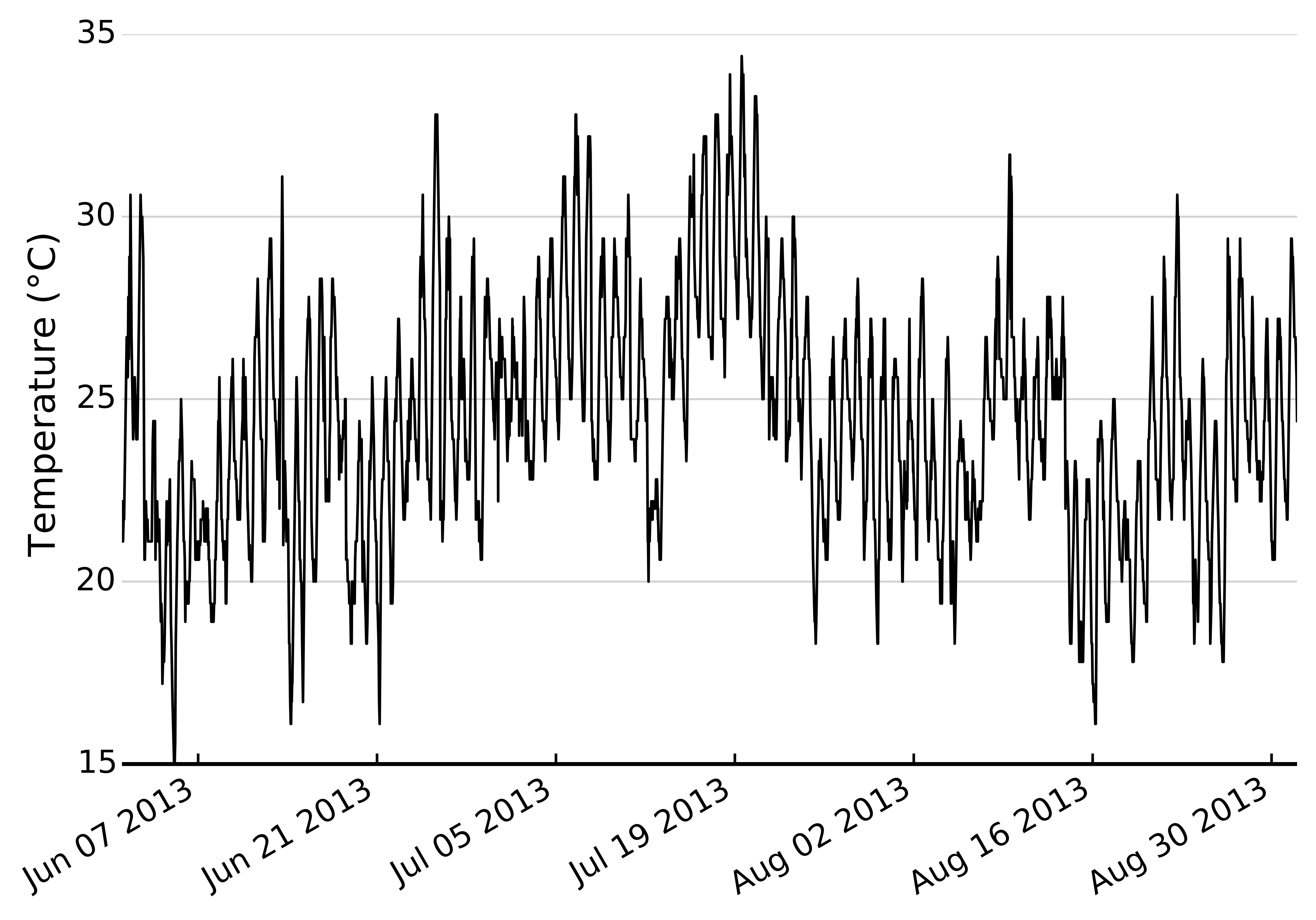

Supplement: Figure S2 [file peerj-02-376-s002.png]
