# Supplementary material for: Variable effects of temperature on insect herbivory
Source: PeerJ. 2014 May 6;2:e376. doi: 10.7717/peerj.376 (PMC4017821; doi:10.7717/peerj.376)
Supplement: Table S1 [file peerj-02-376-s003.docx]

| **Herbivore** | **Plant** | **20˚** | **25˚** | **30˚** | **35˚** |
| --- | --- | --- | --- | --- | --- |
| Unidentified Lepidopteran Leafroller | *Lindera benzoin* | 7 | 8 | 9 | 9 |
| *Arge scapularis* | *Ulmus rubra* | 3 | 2 | 3 | 2 |
| *Atteva aurea* | *Ailanthus altissima* | 3 | 4 | 5 | 4 |
| *Chrysocus auratus* | *Apocynum cannibinum* | 5 | 5 | 5 | 5 |
| *Danaus plexippus* | *Asclepias syriaca* | 3 | 3 | 3 | 3 |
| *Epimecis hortaria* | *Lindera benzoin* | 9 | 9 | 9 | 9 |
| *Epimecis hortaria* | *Liriodendron tulipifera* | 8 | 8 | 8 | 8 |
| *Epimecis hortaria* | *Sassafras albidum* | 4 | 4 | 4 | 3 |
| *Euchaetes egle* | *Apocynum cannibinum* | 4 | 5 | 5 | 4 |
| *Euchaetes egle* | *Asclepias syriaca* | 5 | 5 | 5 | 4 |
| *Hyphantrea cuneata* | *Acer negundo* | 8 | 8 | 8 | 7 |
| *Hyphantrea cuneata* | *Liquidambar styraciflua* | 8 | 8 | 6 | 8 |
| *Malacosoma americanum* | *Prunus serotina* | 4 | 7 | 6 | 6 |
| *Melanophia canadaria* | *Acer negundo* | 4 | 4 | 4 | 4 |
| *Melanophia canadaria* | *Lindera benzoin* | 9 | 9 | 9 | 9 |
| *Melanophia canadaria* | *Sassafras albidum* | 4 | 4 | 4 | 4 |
| *Nematus tibialis* | *Robinia pseudoacacia* | 6 | 5 | 9 | 9 |
| *Papilio polyxenes* | *Foeniculum vulgare* | 5 | 5 | 6 | 4 |
| *Papilio troilus* | *Lindera benzoin* | 5 | 6 | 6 | 6 |
| *Papilio troilus* | *Sassafras albidum* | 6 | 5 | 6 | 5 |
| *Saucrobotys futilalis* | *Apocynum cannibinum* | 9 | 12 | 9 | 9 |
