# Supplementary material for: Variable effects of temperature on insect herbivory
Source: PeerJ. 2014 May 6;2:e376. doi: 10.7717/peerj.376 (PMC4017821; doi:10.7717/peerj.376)
Supplement: Table S2 — Temperature data for each growth chamber, collected by HOBO pendant temperature loggers (HOBO UA-002 pendant loggers, Onset Computer Corporation, Bourne MA). [file peerj-02-376-s004.docx]

| **Set Temperature (˚C)** | **Mean Temperature (˚C)** | **Standard Deviation** |
| --- | --- | --- |
| 20˚ | 19.59˚ | 0.53˚ |
| 25˚ | 26.14˚ | 0.26˚ |
| 30˚ | 28.58˚ | 1.89˚ |
| 35˚ | 34.63˚ | 2.05˚ |
