# Supplementary material for: Variable effects of temperature on insect herbivory
Source: PeerJ. 2014 May 6;2:e376. doi: 10.7717/peerj.376 (PMC4017821; doi:10.7717/peerj.376)
Supplement: Table S3 — Regression equations used to correct for autogenic change for each leaf species, where y is the correction factor and Massi is the initial leaf mass. The response variable is change in leaf weight in the absence of herbivory. [file peerj-02-376-s005.docx]

| **Plant Species** | **Equation** | ***R*^2^** |
| --- | --- | --- |
| *Acer negundo* | $y=-0.01+0.11{Mass}_{i}$ | 0.68 |
| *Ailanthus altissima* | $y=-0.01+0.02{Mass}_{i}$ | 0.61 |
| *Apocynum cannabinum* | $y=0.00+0.09{Mass}_{i}$ | 0.70 |
| *Asclepias syriaca* | $\log(y)=-2.19+0.29{Mass}_{i}$ | 0.28 |
| *Foeniculum vulgare* | $y=0.00+0.16{Mass}_{i}$ | 0.91 |
| *Lindera benzoin* | $y=0.00+0.03{Mass}_{i}$ | 0.41 |
| *Liquidambar styraciflua* | $y=0$ |  |
| *Liriodendron tulipifera* | $y=-0.02+0.07{Mass}_{i}$ | 0.44 |
| *Prunus serotina* | $y=0.01+0.17{Mass}_{i}$ | 0.95 |
| *Robinia pseudoacacia* | $y=0.00+0.07{Mass}_{i}$ | 0.65 |
| *Sassafras albidum* | $y=-0.01+0.08{Mass}_{i}$ | 0.50 |
| *Ulmus rubra* | $y=0.00+0.06{Mass}_{i}$ | 0.57 |
